# Redefining Age-Friendly Neighbourhoods: Translating the Promises of Blue Zones for Contemporary Urban Environments

**DOI:** 10.3390/ijerph21030365

**Published:** 2024-03-19

**Authors:** Peyman Najafi, Masi Mohammadi

**Affiliations:** 1 Chair Smart Architectural Technologies, The Built Environment Department, Eindhoven University of Technology, Vrt 7.29, Groene Loper 3, P.O. Box 513, 5612 AE Eindhoven, The Netherlands; m.mohammadi@tue.nl; 2 Chair Architecture in Health, The Built Environment Department, Han University of Applied Sciences, Postbus 5375, 6802 EJ Arnhem, The Netherlands

**Keywords:** Blue Zones, age-friendly communities, healthy ageing, urban design, convolutional neural networks, systematic literature review

## Abstract

The longevity and healthy ageing observed in Blue Zones offer valuable insights for contemporary urban neighbourhood planning and design. This paper reviews the age-friendly features of the built environment in Blue Zones, aiming to translate these insights into actionable strategies for urban neighbourhood development. Employing a systematic literature review and Convolutional Neural Networks (CNNs) analysis of Google Street View imagery, this study assesses the built environments in Blue Zones, including housing, public spaces, and transportation systems. Key findings highlight the presence of adaptable housing, building designs that foster a connection with nature, and semi-public spaces that encourage social interaction and physical activity. A notable emphasis on walkability and limited public transport access was observed. The CNN analysis corroborated these findings, providing a quantitative view of age-friendly features. This research contributes to the academic discourse in urban planning and design by providing practical insights for developing age-friendly neighbourhoods, inspired by Blue Zones. It concludes by offering policy advice and future research directions for creating sustainable and inclusive urban environments conducive to ageing populations.

## 1. Introduction

The World Health Organisation (WHO) strives to assist and inspire contemporary urban environments to become more ‘age-friendly’ through the Global Age-Friendly Cities and Neighbourhoods Guide [1]. An age-friendly neighbourhood offers a supportive environment that enables residents to grow older actively within their families and civil society and offers extensive opportunities for their participation in the community [2]. In attempts to make contemporary urban environments more age-friendly, the concept of Blue Zones—regions of the world where people live exceptionally long lives—offers a fascinating lens for urban planners, designers, and neighbourhood policymakers [3,4,5,6]. These zones are renowned for their unique lifestyle and environmental characteristics known to contribute to longevity and healthier ageing [7,8,9]. Understanding these characteristics in urban planning and design is critical for shaping sustainable, accessible, and engaging urban environments tailored for ageing populations. As global demographics increasingly shift towards an older age spectrum, the need for adaptable, age-friendly urban environments becomes more pressing [10,11]. Examining Blue Zones allows urban planners and designers to glean valuable lessons on enhancing the quality of life for senior citizens within urban environments. This paper aims to systematically review the age-friendly features of the built environment in Blue Zones, employing a combination of qualitative and quantitative approaches to provide comprehensive insights and neighbourhood development policy advice.

The concept of Blue Zones was introduced to the academic community nearly 20 years ago. Michel Poulain and Giovanni Mario Pes coined the term following their demographic research in the Nuoro province of Sardinia, Italy, where they noted an exceptionally high number of centenarians [12]. This discovery was focused on a cohort of centenarians residing in 14 mountainous villages of Sardinia. Researchers devised the Extreme Longevity Index (ELI), calculated as the centenarian count per 10,000 newborns. Their analysis revealed an average ELI of 508 per 100,000 births, a figure notably two to four times higher than the ELI values observed across the remainder of Sardinia. Expanding on this research, Dan Buettner identified four additional regions with similarly elevated centenarian populations [13]:

- Okinawa, Japan, is renowned for having the world’s highest life expectancy among women [14,15,16]. The Centenarian Rate (CR)—the proportion of those surviving to age 100 per 10,000 individuals alive at 60—for men is markedly higher compared to those observed in other regions, while the rates for women are typically higher than or equal to those found in other regions [8].
- The Nicoya Peninsula in Costa Rica is distinguished by the Vienna Yearbook of Population Research as a longevity hotspot. A survival analysis from 1990 to 2011 of 1630 older adult Costa Ricans revealed a male death rate ratio (DRR) in Nicoya of 0.80 (95 percent CI: 0.69–0.93). For Nicoyan males aged 60, the probability of reaching centenarian status was sevenfold that of their Japanese counterparts, accompanied by a life expectancy surplus of 2.2 years [17,18].
- Ikaria, Greece, a small island in the Aegean Sea, boasts a significant centenarian population [19,20,21]. Cardiologic examinations conducted in Ikaria in 2009 highlighted the anomalously high average age at natural death, which surpasses that of other global regions, including Greece, by nearly a decade, with over 30% of fatalities occurring post-90 years of age [13].
- Lastly, Loma Linda in Southern California, USA, with its large Seventh-Day Adventist community, has a life expectancy nearly a decade longer than the average American [22].

**Figure 1:**
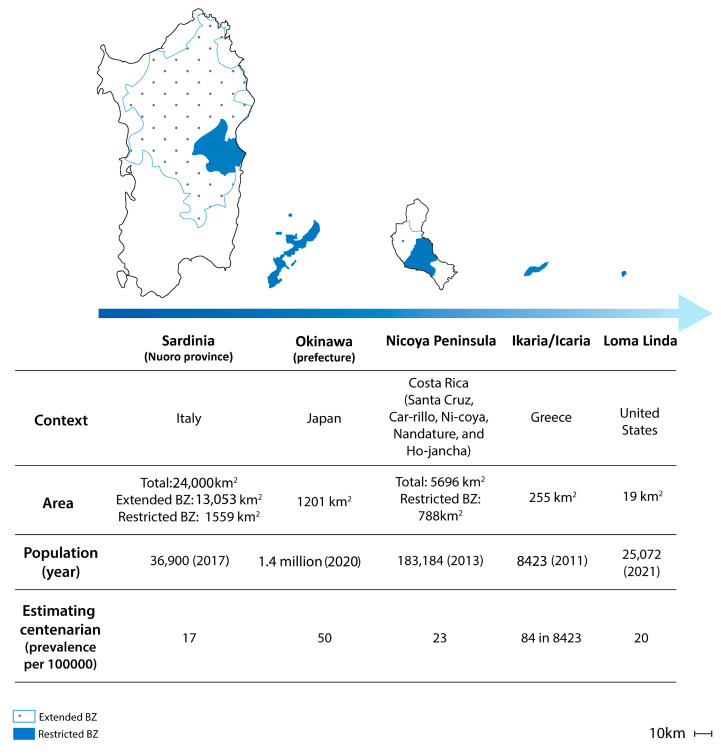
The key demographic and spatial factors of Blue Zone local representatives; source: authors.

Figure 1 summarises the key demographic and spatial factors of local representatives of Blue Zones.

While extensive epidemiological research has explored various facets of Blue Zones, such as diet [22,23], mental health [18,24,25], cardiovascular health, longevity, obesity, and physical activity, a notable gap persists in understanding the contribution of urban planning and design to these exceptional health outcomes [4,6]. This study aims to examine the specific elements of the built environment that potentially underpin the success of Blue Zones. This inquiry holds particular significance for the field of urban planning and design, especially in the context of neighbourhood planning and policymaking. A primary aim of this research is to develop practical guidelines and policy advice, especially in understanding how the built environment in Blue Zones promotes healthy ageing among its residents.

To achieve this, we will conduct an in-depth review of relevant research and perform a spatial analysis of built environments in Blue Zones to derive comprehensive recommendations and neighbourhood development policy advice.

This review will collate insights from existing research on the built environment characteristics of Blue Zones. Next to this, an in-depth spatial analysis is essential for an empirical examination of the built environment features in Blue Zones. However, the global dispersion of Blue Zones makes traditional methods like field surveys challenging due to the extensive time, cost, and effort involved. Fortunately, recent advancements in AI and deep learning algorithms, particularly Convolutional Neural Networks (CNNs), offer a viable alternative. These technologies allow for the remote, high-level analysis of digital images. In our study, CNN techniques are employed to analyse the built environment features of Blue Zones by using publicly available datasets, such as Google Street View (GSV). This approach provides empirical evidence to supplement our qualitative literature review, enhancing our understanding of the age-friendly built environment features in Blue Zones.

The structure of this paper is as follows: Section 2 provides an overview of the variables that contribute to an age-friendly built environment. Understanding these variables is crucial, as it guides our exploration of specific built environment features in the Blue Zone literature review and the CNN analysis. Section 3 details our methodology, elaborating on how we structured our systematic literature review and CNN analysis to investigate age-friendly built environment features in Blue Zones research and the GSV imagery. Section 4 presents our findings, culminating in insights for Blue Zone Neighbourhood (BZN) policy advice, aimed at enhancing healthy ageing within contemporary neighbourhood planning and design initiatives. In Section 5, we discuss the implications of our findings, reflecting on how they contribute to existing knowledge and suggesting potential implications for future research in urban design and planning. Finally, Section 6 concludes the paper, summarising our key findings and the future direction of our research for shaping healthy ageing neighbourhoods within contemporary society.

## 2. Materials: Age-Friendly Built Environment Variables

The built environment encompasses human-crafted spaces for dwelling, working, and leisure activities. The WHO champions age-friendly built environments as part of its Global Age-Friendly Cities (AFC) initiative [1,11,26]. This concept includes three built environment domains: housing and buildings, open/outdoor public spaces, and transport networks.

### 2.1. Age-Friendly Housing and Buildings

Providing adequate housing that enables older adults to age in their home environment while retaining their autonomy and independence is pivotal for promoting an age-friendly built environment [27,28]. Central to this is the ‘ageing in place’ concept, which allows older individuals to remain in their homes, living independently and comfortably, irrespective of age, income, or abilities [29]. This approach goes beyond staying in the same location; it encompasses adapting to or relocating to more suitable environments as needs evolve. Attachment to place plays a significant role, fostering a sense of identity and independence [30,31]. Ageing in place might involve staying in the same home or transitioning to more accessible residences, facilitating independent or assisted living [28].

Housing options for older adults vary depending on their mobility and health needs, ranging from regular houses and apartments to retirement communities for independent living, as well as specialised care homes offering intensive support [32]. This spectrum includes various housing typologies alongside institutional care facilities for highly dependent individuals. Ageing in place can delay or eliminate the need for institutional care [28].

The design of housing with ageing needs in mind is critical to ensure that older adults can comfortably and safely age in place while maintaining their independence and well-being [23]. Such designs enable accessibility and ease of movement for residents of varying abilities, minimising later modifications. Age-friendly homes benefit not only the residents but also the wider community by reducing reliance on institutional care facilities [27]. Alongside the design requirements, the buildings’ facilities, particularly public ones, must be accessible and user-friendly for all ages and abilities. Essential features include ramps, elevators, handrails, anti-slip floors, seating, accessible toilets, clear signage, and designated parking areas [1,4,31].

### 2.2. Age-Friendly Open/Public Spaces

An age-friendly built environment is also associated with neighbourhoods that accommodate older adults, featuring easily accessible public/open spaces, supportive social services, and inviting third places, which can substantially improve life quality and overall well-being for this demographic [32]. Easily accessible spaces mainly refer to those public spaces that promote safety and comfort and encourage outdoor engagement among older adults [33]. As loneliness and depression are prevalent among older adults, particularly those living independently without spousal or partner support, accessible open spaces enable them to socialise and age within their neighbourhoods [34]. Common areas such as coffee shops, public gardens, retail centres, libraries, marketplaces, and local community hubs are considered as alternative social spaces, separate from homes and workplaces [35]. These spaces, also well known as “third places”, can foster voluntary social interaction and provide opportunities for older individuals to be involved in their communities and build social networks, both casual and close, which are essential in combating solitude and isolation [36,37].

Open spaces accommodating older adults are also linked to encouraging physical exercise and improving their health [38,39,40]. Regular physical activities, especially walking, which is a preferred exercise among older adults, are facilitated by neighbourhoods designed to be pedestrian-friendly. This is mainly characterised by a balanced mix of different land uses, well-connected pathways, and dense residential areas [41,42]. Mixed-use developments, with their integration of residential, commercial, and communal spaces, attract people outdoors and encourage regular walking. The layout of streets and the size of blocks play a crucial role in how accessible a neighbourhood feels. The number of people in an area contributes to a dynamic and engaging urban life, creating a sense of belonging in the community. Older adults residing in areas with varied land uses, interconnected streets, and dense populations are more inclined to be physically active, owing to the closeness of various destinations [43]. Other factors that affect the walkability of the built environment include pedestrian safety from vehicular traffic with lower speed limits and more road crossings, the connectivity of pedestrian facilities, public safety from crime and violence, trees and vegetation for shading and amenities, adequate lighting for illumination, distinctive signage for wayfinding, user-friendly street furniture, and inclusive urban designs with barrier-free access [44,45].

### 2.3. Age-Friendly Transportation Systems

Neighbourhoods designed for walking prioritise a secure, interconnected network for pedestrians, blending different modes of transport to support the mobility needs of older adults. Consistent, dependable, and secure public transportation is crucial in facilitating the ease of navigation for seniors in their environment. The quality of services and perceived travel safety significantly influence older adults’ perceptions of public transit accessibility [46]. Age-friendly public transportation facilities, such as train stations, tram stops, and bus stops, are equipped with comfortable seating, shelters, and adequate lighting, creating an inviting environment for older travellers. Ramps, elevators, escalators, and low-floor boarding onto buses enhance accessibility for seniors, allowing them to confidently utilise public transport. Priority seating on public transport encourages passengers to offer seats to older adults and others in need, promoting inclusive and considerate travel experiences. Cost-effective public transportation is also a key element in maintaining mobility for the elderly. In many countries, older adults are offered concession fares or even free access to public transportation, such as buses, trains, and trams [47]. Improved mobility through public transport not only enhances the quality of life of older adults but also contributes to their social inclusion and engagement [48].

Beyond the ease of walking, evaluating neighbourhood transportation also involves considering its suitability for cycling, often referred to as its bikeability or the degree to which it is bicycle-friendly [49]. The act of bicycling serves as a green method of transportation, contributing to the reduction in vehicle use and the enhancement of air quality. Moreover, it acts as a physical activity, aligning with the goals of active ageing and boosting public health [50]. The participation of older adults in cycling is influenced by a range of factors, including the separation from motor traffic, safety concerns, the behaviour of fellow cyclists, and the ease and presence of necessary infrastructure [51,52].

It is also noted that senior citizens residing in suburban and remote locations, where public transportation is scarce, often find their ability to move around and their overall well-being tightly linked to owning a vehicle. As they age, a gradual reduction in cognitive, sensory, and physical capacities can lead to slower reflexes, diminished vision, impaired hearing, challenges in focusing, and memory issues. These changes can increase the risks associated with driving and raise various safety concerns [53,54].

In summary, to provide a structured understanding of age-friendly built environment variables and their associated features, we compiled them into Table A1, which is presented in Appendix A. This table serves as a guiding framework throughout this study, particularly in analysing the alignment of Blue Zones with these age-friendly built environment standards. The subsequent sections will refer to this table to assess the built environments in Blue Zones, integrating insights from both the systematic literature review and the CNN analysis.

## 3. Methodology

**Figure 2:**
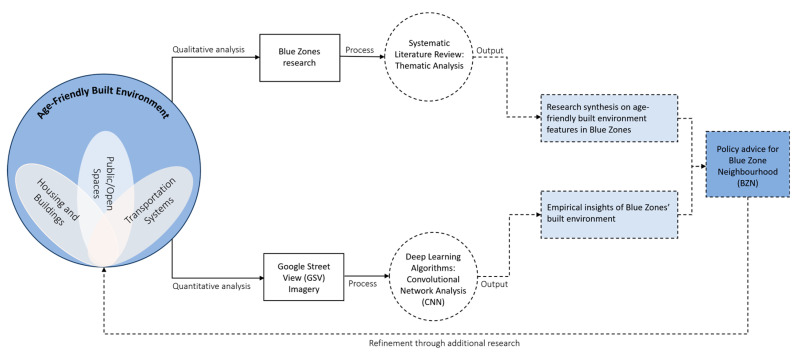
The exploration framework for examining age-friendly built environment features in Blue Zones. Details of the age-friendly built environment variables, along with their contributing descriptions, are provided in Appendix A. Dashed lines within the framework indicate areas for ongoing research and potential refinement.

In this research, we adopt a dual-method approach, integrating both qualitative and quantitative analyses, to thoroughly investigate the age-friendly aspects of the built environment in Blue Zones. Our methodology includes a systematic literature review and a CNN analysis, each providing unique insights into the age-friendliness of these environments. The methodological framework, depicted in Figure 2, illustrates the progression of our study from reviewing the existing literature and conducting a deep learning neural networks analysis to generating new insights and policy advice for contemporary urban neighbourhood initiatives. This approach can contribute to the existing body of knowledge by leveraging innovative methods to enhance our understanding of built environments within urban studies. The framework acts as a guiding roadmap for our analysis, informing our examination of the Blue Zones and our contributions to urban neighbourhood planning and policy development. We will delve into the details of this framework in the subsequent sections.

### 3.1. Systematic Literature Review: Thematic Analysis

Among several approaches suitable for conducting a qualitative analysis, we selected a thematic analysis as it allows us to identify, analyse, and interpret patterns of meaning within qualitative datasets [55]. Within our study, a thematic analysis will help us to unravel and interpret patterns in the Blue Zones research relevant to (1) housing and buildings, (2) public/open spaces, and (3) transportation systems. The interpretations can later help us to develop possible policy advice and recommendations by illuminating the interconnectivity of themes and their implications for neighbourhood design and planning.

#### 3.1.1. Summary of Search Strategy

We carefully selected the relevant academic sources by using defined criteria such as keyword relevance, language, and publication date. Online databases including Scopus, Google Scholar, Web of Science, and PubMed, along with a manual snowball search—identifying related works by consulting the bibliography or references section at the conclusion of a paper—completed on 15 September 2023 provided a comprehensive collection of the international literature. Peer-reviewed papers, book chapters, and grey literature such as reports and conference proceedings were reviewed for inclusivity.

#### 3.1.2. Study Selection

**Figure 3:**
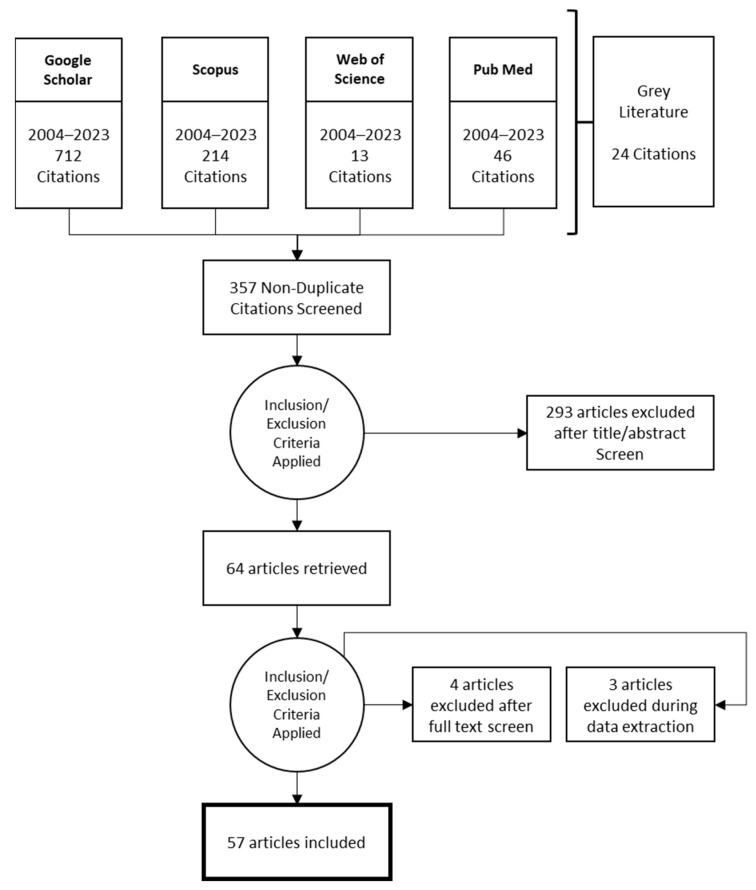
Selected papers for thematic analysis using PRISMA.

In online databases, “Blue Zones” is used as the primary keyword in the search within titles, abstracts, and keywords. Additional terms “Sardinia”, “Okinawa”, “Ikaria”, “Nicoya Peninsula”, “Loma Linda”, and “Adventist” are also considered based on their frequency in the retrieved articles. To broaden the search, synonyms of the term Blue Zone such as “longevity hotspots” and “world’s longest-lived populations” are included (Figure 2). The search timeframe spans from 2004 to 2023, coinciding with the emergence of the Blue Zones concept [12]. Non-peer-reviewed papers (e.g., reports, abstracts, book chapters, and conference proceedings) and selected papers were included separately (category “grey literature”) and used to hand-select additional relevant resources. An initial inquiry resulted in 357 non-duplicated citations for further investigation (Figure 3).

#### 3.1.3. Inclusion and Exclusion Criteria

All 357 non-duplicated citations were screened by using the title and abstract. The selected papers met the following criteria:

- Geographic location of study: the research focused on the five established Blue Zones.
- Exposure of interest: the paper targeted populations of centenarians and older adults aged 75 years and older. Language: published in English between 1 January 2004 and 19 September 2023.
- Peer review: the paper was peer-reviewed.
- Reported outcomes: The outcome of the paper reported the influential factors of the longevity of centenarians in Blue Zones. Selected papers were examined on whether influential factors were reported appropriately and in a consistent manner. Papers/sources using self-reported outcomes rather than objective measures were excluded.
- Prior to final selection, it was deemed necessary to exclude Loma Linda from the study due to its distinct contextual differences compared to other regions. Due to distinct contextual differences, Loma Linda was excluded from this study. This decision was based on its unique demographic and cultural landscape, the quality and availability of healthcare services, and its economic landscape, which differs significantly from the other Blue Zones. Additionally, Loma Linda’s level of urbanization and environmental factors diverge from the more isolated and less urbanised settings of other Blue Zones.

Ultimately, this review encompasses 57 peer-reviewed publications, forming the basis for an in-depth thematic analysis detailed in Appendix B.

While the systematic study selection provides a rich repository for qualitative analysis, we acknowledge some limitations: This research was also restricted to publications available in English, thus potentially excluding valuable content in other languages from the interpretation. Additionally, by exclusively focusing on online resources and full-text publications, there is a possibility of overlooking the most recent evidence, such as abstracts, which may provide valuable insights. To mitigate this, we included a thorough review of bibliographies to encompass a broader range of perspectives.

Finally, to delve deeper into our qualitative data, we utilised ATLAS.ti 23 for a thorough thematic analysis. This involved meticulously coding and categorising the data, ensuring alignment with the age-friendly built environment variables that we previously identified and listed in Appendix B.

### 3.2. CNN Analysis

To complement our qualitative analysis, we deployed a deep learning neural network, specifically a CNN, capable of processing assembled data and extracting key features [56]. In our study, the CNN primarily detects age-friendly built environment variables within the GSVs, providing quantitative support for our qualitative findings (details in Appendix C). Additionally, the CNN analysis employs image segmentation techniques to classify the object types associated with these variables. This method involves segmenting and categorising different areas within the images, allowing for a more precise and detailed analysis and interpretation of each object [57].

#### 3.2.1. GSV Imagery Acquisition

**Figure 4:**
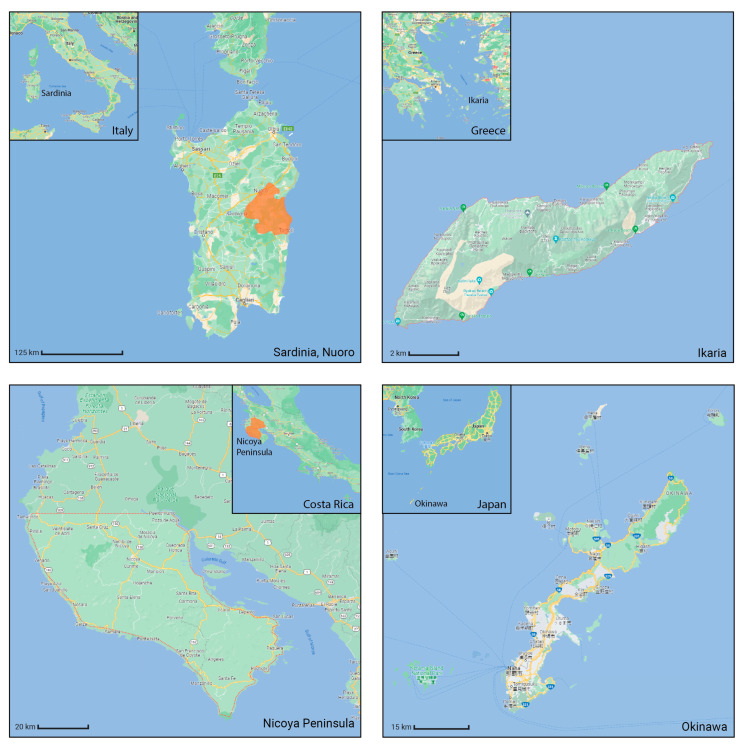
The four Blue Zones located in Sardinia, Ikaria, Costa Rica, and Okinawa are illustrated in accordance with the geographical delineations provided by [58].

To initiate the CNN analysis, we first needed to collect relevant images of the built environment in Blue Zones from various open datasets known for offering comprehensive urban imagery. Among these, we employed the Google Street View (GSV) platform, which is recognised in urban studies for its extensive and regularly updated street-level imagery. We utilised the GSV API to systematically retrieve images of Blue Zones from Google Services. We delineated the boundaries of the Blue Zones in accordance with Poulain’s demographic study, in which he and his team identified regions inhabited by the longest-lived populations [7] (Figure 4).

In acquiring GSV imagery, several criteria were tailored to effectively facilitate the CNN analysis. These criteria included the density of the GSV imagery, the diversity of the built environment, and the computational capabilities of our model. Adjustments to the GSV API parameters were made to optimise the image-capture process. Specifically, we set the heading to 180 degrees to standardise the image direction, adjusted the pitch to 0 degrees to align the camera angle horizontally, and set the field of view to 50 degrees to ensure a wide yet detailed view. Each image was obtained at a resolution of 600 × 600 pixels, which is the highest resolution available through the GSV API.

#### 3.2.2. Pre-Processing

After collecting the Blue Zones GSV imagery, our initial step involved reviewing and curating the images, removing those that were not relevant or of poor quality. Ultimately, a total of 2980 images were selected for the final dataset, which we divided into three parts:

- The training set (70%) is used to train the CNN model, enabling it to recognise patterns within the data.
- The validation set (15%) is used not for training but rather to tune hyperparameters and provide an unbiased evaluation of the model fit during the training phase.
- The testing set (15%) is employed post-training and validation to offer an unbiased evaluation of the final model’s performance.

During the collection of the GSV imagery, we encountered significant variations in image availability across the different Blue Zones. The GSV coverage for Okinawa and Sardinia was nearly comprehensive, providing a rich dataset for these regions. Conversely, the availability in Ikaria and the Nicoya Peninsula was more limited, reflecting variations in geographical accessibility and Google’s mapping efforts. To address these discrepancies, we made conscientious efforts to maximise the utility of the available data, ensuring our analysis is as representative and comprehensive as possible within the existing constraints. Overall, the final dataset comprised 2086 GSVs for training, 447 GSVs for validation, and 447 GSVs for testing our model. Table 1 details the number of GSVs selected for each class in our dataset.

#### 3.2.3. CNN Model Architecture and Object Extraction

Our CNN model employs the YOLO (You Only Look Once) architecture developed by Redmon et al. (2017) [59]. This architecture is renowned for its efficiency and accuracy in object detection across diverse contexts [60]. We selected YOLOv5 for its proven efficacy in urban design applications, as evidenced by [61,62,63]. YOLOv5 was instrumental in identifying and extracting objects from the Blue Zones’ GSV imagery.

A crucial aspect of developing and validating object-detection models is image annotation, which entails labelling or classifying objects within images. For this purpose, our study employed the ADE20K dataset [64], a widely acknowledged resource in urban scene parsing. This dataset facilitated the annotation of age-friendly built environment features in the Blue Zones’ GSV imagery, and the process is elaborated upon in Appendix C.

### 3.3. Model Performance Evaluation

We evaluated our CNN model by using four key evaluation metrics, namely Mean Average Precision (mAP), Precision, Recall, and Intersection over Union (IoU):

- Mean Average Precision (mAP): This metric measures the accuracy of an object-detection model. A higher mAP value indicates better performance. Specifically, it is calculated by averaging the Precision scores across all classes at different IoU thresholds. In the case of YOLOv5, the average Precision is calculated at an IoU threshold of 0.5.
- Precision: This metric reflects the proportion of correctly predicted positive observations to the total predicted positives. It is defined as the number of true positives (TP, or correctly identified positive instances) divided by the sum of true positives and false positives (FP, or negative instances incorrectly identified as positive):
- Recall (Sensitivity): Recall measures the model’s ability to correctly identify all actual positives. It is calculated as the number of true positives divided by the sum of true positives and false negatives (FN, or positive instances incorrectly identified as negative):
- Intersection over Union (IoU): IoU is a metric used in object detection to evaluate the accuracy of a predicted bounding box. It calculates the area of overlap between the predicted bounding box and the ground truth bounding box, divided by the area of union between these two boxes:

These metrics collectively provide a comprehensive evaluation of our CNN model’s performance in detecting and classifying objects within the dataset.

## 4. Results

### 4.1. CNN Model Performance

**Figure 5:**
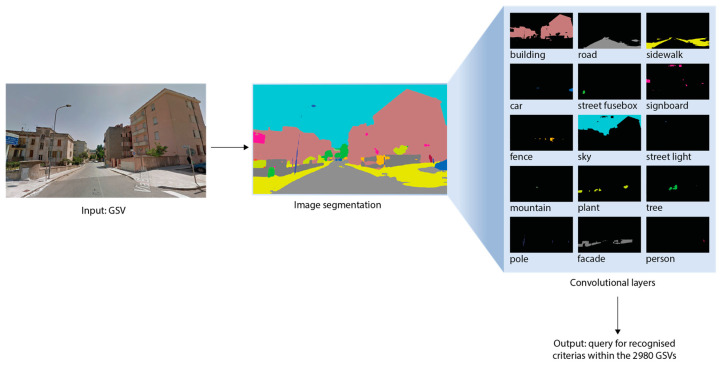
GSV transformation into convolutional layer for CNN image segmentation.

The performance of our CNN model demonstrates its effectiveness in accurately identifying and analysing age-friendly built environment features within Blue Zones GSV imagery. The model achieved a mAP score of 78%, which represents a relatively satisfactory level of accuracy in object detection. The Precision of the model was recorded at 75%, indicating the proportion of true positive detections relative to the total number of predicted positives (true positives and false positives). This highlights the model’s reliability in making predictions. The model also exhibited a Recall rate of 72%, illustrating its capability to correctly identify all actual positives. Furthermore, the IoU score was calculated to be approximately 70%, confirming the model’s effectiveness in localising objects accurately. Figure 5 illustrates the process by which GSV images are transformed into convolutional layers for CNN image segmentation. This transformation subsequently facilitates the identification of the pertinent built environment criteria within a dataset of 2980 GSVs. A detailed analysis of these outputs within the framework of thematic investigation is presented in the ensuing sections.

### 4.2. Blue Zones’ Housing and Buildings

#### 4.2.1. Ageing in Place

Our thematic analysis underscores a strong focus on ageing in place within Blue Zones, deeply influenced by solid social support networks. A notable example of this is seen in Okinawa, where ‘Moai’ groups—social collectives dedicated to providing lifelong support—are prevalent. These groups are instrumental in motivating older centenarians to remain within their communities, helping them to stay actively engaged and connected. In areas such as Sardinia and Nicoya, it is customary for senior individuals to live close to or with their families, ensuring they receive the necessary support [21,65]. This practice is mirrored in Okinawa, where the Moai members often reside within walking distance of each other, fostering consistent support that is especially crucial for ageing in place [5,66].

#### 4.2.2. Housing Options

Our CNN model analysis reveals varied housing options across Blue Zones. In Ikaria, traditional stone houses, villas, and seaside bungalows dominate, with apartment complexes found primarily in central villages. Sardinia displays a wide range, including traditional stone houses (24% of the GSV imagery), modern apartments (62%), luxury villas (9%), and seaside cottages (5%). Okinawa’s landscape comprises modern apartments and condominiums (56% of the GSV imagery), with the rest being stand-alone houses, including Minka and ryokans. In Nicoya, single-story buildings predominate (78% of the GSV imagery), with a mixture of apartments and housing complexes (Table 2).

#### 4.2.3. Building Design

The GSV imagery of the Blue Zones conveys a predominant theme of “simplicity” in housing and building designs. In Ikaria, 95% of the GSV imagery features homes made of stone and natural elements, exemplifying a minimalist approach. This local ethos is also captured in an Ikarian saying [67]: “Home as much as you can fit and place as much as you can afford.” Sardinia’s historical urban areas also reflect simplicity through traditional stone masonry structures. Conversely, Okinawa prioritises functionality with buildings primarily made from concrete or cinder blocks, as observed in over 85% of the GSV imagery.

Moreover, the multifunctionality of living spaces seems to be a key feature across the Blue Zones. Homes often serve as both living- and workspaces, as seen in Ikaria’s flat roofs and Sardinia’s home gardens, which facilitate leisure and family gatherings [68]. In Okinawa, many buildings’ ground floors have dual residential and commercial uses.

Furthermore, the housing design often mirrors a deep connection with nature. In Nicoya, vernacular architectural styles using local materials are set amidst lush greenery and harmony with nature. Even in Sardinia’s more urbanised areas, natural elements are often integrated into Mediterranean-style homes.

Table 2 provides a detailed analysis of age-friendly housing and building features across Blue Zones.

**Table 2:** Age-friendly housing and building features across Ikaria, Sardinia, Okinawa, and Nicoya using CNN evaluation of GSV imagery.

Housing and Building Features		Sampled GSVs
Housing options		-
(a) *	Single-story buildings, apartments, housing complexes	
(b) *	Stone houses, villas, seaside bungalows	
(c) *	Stone houses, modern apartments, villas, seaside cottages	
(d) *	Modern apartments, condos, stand-alone houses (Minka, resort-style villas, ryokans)	
Spatial distribution of buildings		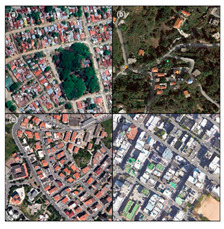
(a)	Null **	
(b)	Sparse and organic settlement patterns, spread out houses	
(c)	Compact space organisation with narrow pathways, limited pedestrian/vehicular circulation	
(d)	Compact with narrow pathways; spacious buildings along central regions	
Dominant building design characteristics (percentage of GSV imagery)		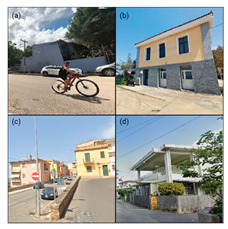
(a)	Vernacular buildings (single-story 78 percent, others 22 percent)	
(b)	Natural elements, Mediterranean architecture (95 percent)	
(c)	Traditional masonry (24 percent), apartments (62 percent), villas (9 percent), cottages (5 percent)	
(d)	Modern-style apartments (56 percent), stand-alone houses (44 percent)	
Dominant design materials		-
(a)	Corrugated metal, bricks, concrete blocks, local materials	
(b)	Stone, natural elements, vegetation incorporated	
(c)	Stone, terracotta, plaster, brickwork, commercial/residential ground floors	
(d)	Concrete, cinder block, minimal ornamentation	
Dominant building layout		-
(a)	Null	
(b)	Layout seems adaptable to sloped terrain	
(c)	Null	
(d)	Null	
Dominant ground floor functionality		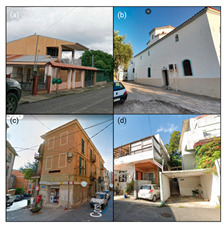
(a)	Null	
(b)	Null	
(c)	Often used for garages or commercial purposes	
(d)	Partly residential, partly for vehicular storage or commercial spaces	
Façade colour		-
(a)	Null	
(b)	White/bright	
(c)	Varied: plastered to exposed brickwork and stone	
(d)	Null	
Roof Style		-
(a)	Null	
(b)	Flat/low-pitched	
(c)	Pitched roofs with terracotta tiles	
(d)	Null	
Integration of Vegetation		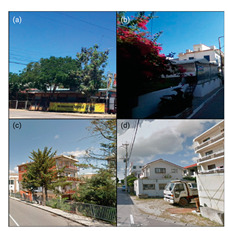
(a)	Null	
(b)	Integrated among buildings as trees and small gardens	
(c)	Null	
(d)	Null	

*: (a): Nicoya Peninsula, (b): Ikaria, (c): Sardinia, (d): Okinawa. **: Insufficient data.

### 4.3. Blue Zones’ Open/Public Spaces

#### 4.3.1. Outdoor Space Accessibility

Our research reveals that Blue Zones commonly feature landscapes that seamlessly integrate with nature. Studies support the notion that living in these environments has a positive impact on the health of older adults [12,21,23,69], (Ref. [70], p. 201). The CNN analysis shows that 65.1% of Blue Zone areas are characterised by open, natural spaces. Ikaria, Sardinia, and the Nicoya Peninsula in particular showcase extensive accessibility to natural landscapes in about 35% of GSVs. Okinawa differs slightly, with 21% of GSVs depicting more urbanised parks and plazas, whereas Sardinia presents a mixture of both natural settings and designed open spaces.

#### 4.3.2. Third Places

In Okinawa, our analysis found a prominent presence of third places such as restaurants and markets, making up about 65% of the GSV imagery. Sardinia also displays a significant number of such places, mainly concentrated in urban centres and residential areas, appearing in 18% of GSVs. Due to limited GSV coverage, the findings in Ikaria and Nicoya are less definitive, but trends in thematic analysis suggest a focus on home-centred social gatherings. These gatherings, often facilitated by family and close-knit groups, are vital in fostering community bonds among older adults [7,8]. Research emphasises the importance of these local social interactions in enhancing community well-being [18,71]. Community events, ranging from shared meals to cultural festivals, are a staple in Blue Zones and are typically accessible and affordable [6]. Semi-public spaces like terraced gardens in Ikaria and residential gardens in Sardinia likely serve as third places for these activities [20,72].

#### 4.3.3. Pedestrian Infrastructure and Accessibility

Our model indicates that Okinawa and Sardinia have robust pedestrian infrastructure, with 39% and 26% of GSVs, respectively, showing pedestrian-friendly features like separated pedestrian routes, crossings, etc. Physical activities such as walking and cycling are less prevalent, noted in a smaller fraction of GSVs. The analysis of Ikaria was limited due to model constraints, leading to an underrepresentation of these activities. In terms of wayfinding, Sardinia and Okinawa show higher instances of signage. Conversely, Ikaria and Nicoya display limited wayfinding aids, mostly around tourist areas. The distribution of street furniture also varies, with a more consistent presence in urbanised areas of Sardinia and Okinawa, indicating a higher integration of urban amenities. This contrasts with the more sporadic distribution in Ikaria and Nicoya [21].

Table 3 overviews our findings on age-friendly features in the open/public spaces of Blue Zones, detailing the prevalence and types of outdoor spaces, social support facilities, and pedestrian infrastructure.

### 4.4. Blue Zones’ Transportation Systems

Multiple peer-reviewed publications, including Poulain et al. (2021) and Fastame et al. (2021), highlight the limited accessibility to public transportation and inconsistent transportation infrastructure in Blue Zones [20,25]. These highlights also align with our CNN model analysis. In Ikaria, the model was unable to distinctly identify features of public transportation infrastructure. Conversely, in Nicoya, bus stations were identified in about 2% of the GSV imagery, primarily near main thoroughfares. Sardinia shows a more consistent presence of public transport amenities like bus stops, visible in approximately 14% of the GSV imagery and widely distributed across residential areas. In Okinawa, the CNN model suggests a more extensive public transportation network, including buses, taxis, and ferries, evident in around 23% of GSV imagery.

Our thematic analysis reveals that the remote and mountainous locations of Blue Zones could pose challenges to transportation access and convenience, especially for older adults. For instance, in the northern areas of Okinawa, where many centenarians reside, the scarcity of personal vehicles complicates access to essential services, leading most seniors to rely on personal transportation [73]. Similarly, in Nicoya, reaching medical centres and social support facilities can take up to 40 min by car, which is particularly challenging for seniors or mobility-impaired residents [17].

Furthermore, our analysis suggests that limited public transport in Blue Zones may be a contributing factor to increased walkability in these regions. Notable themes identified include “increased physical activity”, “cardiovascular health”, “exercise”, and “improved BMI”. Despite a scarcity of dedicated pedestrian paths, the CNN model reveals the prevalence of naturally designed walkways. These pathways, often sloped due to the mountainous terrain, are likely to positively influence residents’ cardiovascular health.

In terms of cycling, the GSV imagery reveals a notable presence of bike tracks and cycling trails in Okinawa, accounting for nearly 19% of the GSV imagery. Conversely, in Sardinia and Nicoya, cycling is less prominent but still present, with about 2% and 1% of the GSV imagery, respectively, showing individuals cycling. Notably, our model did not identify a distinct cycling infrastructure in Ikaria.

Table 4 elaborates on our findings, providing an overview of the transportation features in Blue Zones.

### 4.5. The BZN Policy Advice

After doing an in-depth qualitative analysis of Blue Zones research and a CNN-based examination of relevant GSV imagery, we formulated the Blue Zone Neighbourhood (BZN) policy advice. This advice aims to translate our findings from Blue Zones into actionable strategies for urban design and planning.

As delineated in Table 5, the BZN policy advice encompasses insights aligned with three core age-friendly built environment variables. Each variable in the table is supported by findings from our thematic and CNN analyses of the Blue Zones studies. These policy recommendations are intended for testing in later research phases, aiming to create man-made, age-friendly environments reminiscent of those found in Blue Zones within contemporary urban neighbourhoods.

## 5. Discussion

This study embarked on an exploratory journey to reassess the longevity features of Blue Zones through the lens of age-friendly built environment variables. Our primary ambition was to enrich contemporary urban neighbourhood planning and design with actionable policy advice. By drawing from the unique paradigms of Blue Zones, we aim to enhance environments conducive to ageing populations. Employing a dual-method approach, we merged thematic analysis with deep learning image processing. This combination provided a comprehensive understanding of the built environment within Blue Zones. Our findings offer a new perspective on the integration of age-friendly principles in urban planning and the potential for these principles to be applied universally yet adapted locally.

### 5.1. Key Findings and Their Implications

The formulation of the BZN policy advice, detailed in Section 4.5, stands as a testament to this endeavour. Notably, our study’s objective was not to quantify the age-friendliness of Blue Zones per se, but rather to glean insights from these environments that are naturally conducive to healthy ageing, thereby informing current age-friendly initiatives.

Our findings reveal both the congruence and disparity with the WHO’s standards for an age-friendly built environment. The concept of ageing in place emerged prominently in Blue Zones, exhibiting diversity in its forms—from extended cohabitation to proximity to family and tight-knit communities. These instances underscore the potential influence of ageing in place on longevity in these regions.

The delineation of third places in Blue Zones presented an intriguing divergence from standard age-friendly urban models. Contrary to the anticipated abundance of public spaces like cafes and libraries, our CNN analysis uncovered a scarcity of such conventional third places. Yet, this scarcity did not seem to equate to diminished social interaction. Instead, our qualitative insights suggest a robust social well-being, fostered within natural settings and home-based or semi-private spaces.

Moreover, the encouragement of physical activity in Blue Zones, predominantly nudged by geographical features and lifestyle necessities, offers a fresh perspective compared to structured urban exercise programs. This aspect, alongside the observed deficiencies in transportation infrastructure, represents a notable divergence from established age-friendly norms.

### 5.2. Critique and Counterarguments

Recent critiques in the grey literature challenge the validity of longevity claims in Blue Zones, adding a crucial dimension to our discussion [74,75,76]. These critiques highlight potential inaccuracies in data and record-keeping, advocating for a more cautious approach when interpreting studies on longevity in these regions. While our research aligns with the general narrative of Blue Zones as longevity hotspots, we recognise the possibility of such discrepancies. Given these contrasting views, it is essential that our findings are considered with an understanding of the potential data limitations inherent in Blue Zone studies. This perspective is not only academically significant but also has implications for public health policy and lifestyle guidelines. Accurate data collection and analysis are paramount to robustly validate longevity claims. Recognising these challenges is essential for future research, particularly when such studies can significantly influence public health strategies and recommendations based on the lifestyle and environmental factors associated with Blue Zones.

### 5.3. Summary of Key Implications

From this review study, we distil several implications that warrant further explorations in the future, offering opportunities for scholars to delve into more in-depth discussions:

- The importance of adaptability: The diverse forms of ageing in place observed in Blue Zones highlight the significance of adaptable living arrangements. It is crucial to consider how different cultural and geographic contexts influence the preference for living with family, in close-knit communities, or in remote areas. This adaptability is promising to promote longevity and well-being among ageing populations.
- Redefining social spaces (third places): The concept of third places in Blue Zones partially deviates from typical urban standards, focusing more on natural spaces and home-based interactions rather than commercial or public venues. This can suggest that urban planners and designers should be more flexible in defining social spaces, recognising the value of both natural environments and semi-private spaces like home gardens in fostering social interaction.
- Physical activity through environment design: The encouragement of physical activity in Blue Zones is often a by-product of geographical features and lifestyle necessities like farming or husbandry rather than structured exercise programs. This reminds us that urban design can organically promote physical activity through the incorporation of natural landscapes and the encouragement of active lifestyles.
- Challenging conventional age-friendly standards: The divergence from conventional age-friendly standards in transportation and the unique characteristics of third places in Blue Zones can suggest that conventional standards might not be universally applicable. This underscores the importance of (re)considering context-specific solutions in urban planning and design that respond to the unique cultural, geographical, and social aspects of different communities with greater emphasis.

### 5.4. Limitations of the Research

We acknowledge certain limitations during our study: (1) The scope of the literature review: while thorough, our literature review might not have captured every nuance and complexity present in the diverse environments of Blue Zones. (2) Our CNN analysis, focusing on visual aspects of Blue Zones, may not have fully grasped non-visual factors that contribute to age-friendliness, such as community dynamics and social support systems. (3) Inherent biases could exist in our selection and interpretation of literature, influenced by our research focus and preconceptions. (4) Our analysis, both qualitative and quantitative, might have limitations in depth or breadth, impacting the comprehensiveness of our findings.

## 6. Conclusions

This study’s exploration into Blue Zones provides insightful reflections on contemporary age-friendly urban neighbourhood planning and design. We observed that Blue Zones, characterised by their exceptional longevity, offer invaluable lessons in shaping environments conducive to healthy ageing. The fusion of qualitative and quantitative analyses, incorporating a systematic literature review with a CNN analysis of GSV imagery, allowed us to distil the essence of these longevity environments.

The findings of this study led to the formulation of the BZN neighbourhood planning and policy advice. The BZN underlines the promising role of adaptable living arrangements, the importance of redefining social spaces to include natural and semi-private areas, and the necessity for urban designs that organically promote physical activity. These insights challenge conventional age-friendly standards and highlight the importance of context-specific solutions that respect the unique cultural, geographical, and social fabric of communities. As urban planners and designers, these findings compel us to rethink our approaches and adapt our strategies to foster environments that support ageing populations, drawing inspiration from the natural and community-driven paradigms of Blue Zones. Building upon this, our future research will focus on evaluating the feasibility of implementing the proposed policy advice within novel city digital twin models. This will provide a more comprehensive understanding of the challenges and outcomes associated with implementing Blue Zone practices in diverse urban contexts.

In addition to the underlying promises, this study contributes to the broader discourse on urban planning by showcasing innovative methodologies. In particular, by combining deep learning and Convolutional Neural Networking with the qualitative-quantitative review studies, we provide supportive evidence for interpretations and findings, paving the way for further exploration of these techniques in the field.

## Figures and Tables

**Table 1:** The number of GSVs selected for each Blue Zone in the CNN dataset.

Class	GSV Imagery Count (Access: November 2023)
Ikaria	195
Nicoya Peninsula	385
Okinawa	1200
Sardinia	1200

**Table 3:** Age-friendly built environment instances extracted from the Blue Zones GSV imagery.

Feature		Sampled GSVs
Open/outdoor space instance		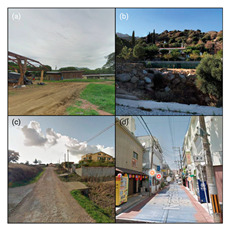
a * (%)	35	
b * (%)	21	
c * (%)	35	
d * (%)	35	
Third place instance		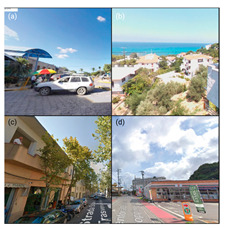
a (%)	18	
b (%)	65	
c (%)	Null **	
d (%)	Null	
Pedestrian infrastructure		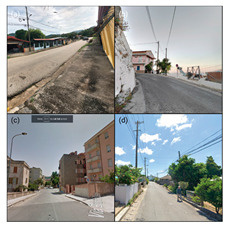
a (%)	26	
b (%)	39	
c (%)	Limited detection	
d (%)	1	
Physical activities		-
a (%)	Walking (7%)	
b (%)	Walking (5%)	
c (%)	Cycling (3%)	
d (%)	Cycling (3%)	
Wayfinding signage		-
a (%)	19	
b (%)	17	
c (%)	2	
d (%)	2	
Street furniture		-
a (%)	55	
b (%)	68	
c (%)	Limited to touristic areas	
d (%)	7	

*: a: Nicoya Peninsula, b: Ikaria, c: Sardinia, d: Okinawa. **: Insufficient data. Note: the percentages indicate the proportion of GSV images in which the respective urban element was identified by the CNN model. “Insufficient Data” denotes areas where the CNN model’s performance was limited due to the sparse availability of GSV images. “Limited Detection” and “Limited to Tourist Areas” refer to observations where the CNN model detected the element in question in a negligible number of images, often confined to specific zones.

**Table 4:** CNN interpretation of Blue Zones’ GSVs focusing on age-friendly transportation systems.

Feature	a ^1^ (%)	b ^1^ (%)	c ^1^ (%)	d ^1^ (%)
Detected public transport modes	Bus stations in 2% of GSVs	Not indicated	Bus or bus stations in 14% of GSVs	Bus or bus stations in 23% of GSVs
Public transport infrastructure	Bus routes near main thoroughfares	Limited bus routes, year-round ferry service	Extensive public transport network with bus stops in residential areas	Superior coverage, frequency, accessibility, and integration
Design in transport hubs	Not indicated	Not indicated	Functional and modern design in 11% of GSVs	Design considers climate; shading in 15% of GSVs, symbolic signage in 65%
Detected bike tracks	1% of GSVs show cycling	None detected	2% of GSVs show cycling	Bike tracks in 19% of GSVs

^1^: (a): Nicoya Peninsula, (b): Ikaria, (c): Sardinia, (d): Okinawa.

**Table 5:** The Blue Zone Neighbourhood policy advice guideline.

Category	Policy Advice
Open/outdoor spaces and neighbourhoods	Maintain scenic outdoor spaces and a symbiotic coexistence with nature as part of neighbourhood planning and design policies.
	Encourage community gardens, urban farming initiatives, and policies supporting local food production and distribution networks within common outdoor areas.
	Promote initiatives encouraging strong social bonds with family members and friends through community programs, events, and shared spaces.
	Foster social networking among neighbours through neighbourhood events, communal spaces, and initiatives encouraging interaction and connection. Maintain a communal social support system that facilitates social connections and assists residents in need through community programs, support groups, and outreach initiatives.
	Integrate physical-activity-friendly design principles in neighbourhood planning, such as walkable layouts, exercise stations, and bicycle infrastructure.
	Incorporate policies that prioritise creating and preserving greenery and protected natural areas within the neighbourhood.
	Promote fair access to parks, recreational facilities, healthcare services, and other community resources, particularly in underserved areas
Transportation systems	Encourage active commuting by integrating and maintaining walking and cycling infrastructure.
	Raise awareness about the health benefits of active commuting and encourage incorporating physical activity into daily travel routines.
	Provide clear signage, wayfinding systems, and information displays for easy navigation and active exploration within the neighbourhood.
	Incorporate accessible pathways with gentle slopes to ensure easy movement.
	Implement safety measures, including well-lit pathways, crossings, and traffic calming strategies.
Housing and buildings	Implement policies to increase the availability and affordability of housing options for low-income individuals and families.
	Encourage the development of housing options that accommodate multiple generations living together, including shared living spaces and adaptable designs.
	Support policies for allocating housing opportunities near family members and loved ones.
	Support policies enabling individuals to live and work in the same space, such as flexible zoning regulations and integrating home office infrastructure.
	Encourage housing designs that maximise space utilisation, offering versatile layouts and adaptable features to accommodate various needs and activities.
	Promote housing designs that incorporate elements reflecting the local culture and blend harmoniously with the natural surroundings.
	Incorporate accessible and well-designed outdoor spaces within housing developments, providing residents easy access to gardens and supporting home gardening initiatives.

## Data Availability

Data is contained within the article.

## Appendix A

**Table A1:** Age-friendly built environment variables: description and examples.

Variable	Description	Examples/Specific Types		
Housing and buildings	Age in place	Attachment to place		
	Variety of housing options	Standard family homes		
		Apartments		
		Retirement institutes		
	Design features facilitating ageing in place	Ramp		
		Elevator		
		Handrail		
		Anti-slip floor		
		Seating		
		Accessible toilet		
		Wayfinding signage		
		Parking area		
Public/open space	Third places	Café, park, shopping mall, library, market, community centre		
	Features encouraging physical activity	Walking	Land use mix	
			Street connectivity	Street pattern
				Block size
			Residential density	Density of concentration of people
		Pedestrian-friendly facility	Tree/vegetation for shading	
			Adequate lighting	
			Pedestrian road	
			Wayfinding signage	
			Street furniture	Seating bench, shelter,
Transportation system	Walkability	Accessibility and convenience	Modes	Walking, bike, bus, tram, train
			Service quality	
			Travel safety perception	
			Design of station	Ramp
				Elevator/escalator
				Low-floor boarding
			Priority seat	
			Affordability	
	Bikeability	Cycling-friendly infrastructure	Segregation from vehicular traffic	
			Safety	
			Cyclist behaviour	
			Convenience	
			Availability of cycling infrastructure	

## Appendix B

**Table A2:** Final dataset selected for the systematic literature review pertaining to Blue Zones.

Topic Area		Authorship	Journal Title	Citation	Publication Year
Sardinia		[12]	Experimental Gerontology	182	2004
		[77]	Journal of Ageing Research	33	2011
		[78]	Biodemography and Social Biology	28	2012
		[79]	Vienna Yearbook of Population Research	2	2013
		[78]	Vienna Yearbook of Population Research	11	2013
		[80]	Journal of Biosocial Science	2	2015
		[81]	European Geriatric Medicine	1	2015
		[82]	Journal of Clinical Gerontology and Geriatrics	5	2016
		[83]	Europe’s Journal of Psychology	15	2017
		[24]	Ageing Clinical and Experimental Research	30	2018
		[84]	Journal of Ageing and Physical Activity	16	2018
		[85]	PLoS ONE	12	2018
		[72]	Behavioral Sciences	14	2018
		[86]	Annals of Medicine	6	2018
		[87]	Ageing Clinical and Experimental Research	8	2020
		[25]	International Psychogeriatrics	11	2021
		[88]	Journal of Religion and Health	5	2021
		[89]	Journal of Ethnic Foods	1	2022
		[90]	International Journal of Psychology	1	2022
		[91]	Journal of Happiness Studies	3	2022
		[68]	Psychology, Health and Medicine	2	2022
Okinawa		[14]	Age (Dordrecht, Netherlands)	63	2006
		[92]	The Journals of Gerontology. Series A, Biological	136	2006
		[93]	Annals of the New York Academy of Sciences	228	2007
		[94]	Journal of Cross-Cultural Gerontology	40	2007
		[73]	The Journals of Gerontology: Series A	59	2008
		[15]	The Journals of Gerontology. Series A, Biological	53	2008
		[70]	Experimental Gerontology	31	2012
		[95]	Experimental Gerontology	7	2013
		[16]	The Journals of Gerontology. Series A, Biological	17	2014
		[96]	Mechanisms of Ageing and Development	19	2017
		[97]	Mechanisms of Ageing and Development	24	2017
Ikaria		[98]	Cardiology Research and Practice	30	2010
		[19]	Cardiology Research and Practice	65	2011
		[99]	Maturitas	49	2011
		[100]	QJM: An International Journal of Medicine	12	2011
		[101]	Maturitas	6	2013
		[102]	International Journal of Cardiology	4	2013
		[103]	Heart and Vessels	13	2013
		[104]	Hellenic Journal of Cardiology	5	2013
		[105]	Angiology	16	2016
		[69]	Hellenic Journal of Cardiology	11	2016
		[21]	International Journal of Environmental Research an	7	2021
		[106]	Hellenic Journal of Cardiology	0	2022
Nicoya Peninsula		[17]	Vienna Yearbook of Population Research	33	2013
		[107]	Experimental Gerontology	45	2013
		[18]	Ageing Clinical and Experimental Research	9	2020
		[108]	Journal of Population Ageing	0	2022
Uncategorised Blue Zone		[8]	American Journal of Lifestyle Medicine	103	2016
		[109]	Mediterranean Journal of Nutrition and Metabolism	11	2018
		[110]	Nutrients	21	2020
		[111]	American Journal of Medicine	1	2020
		[20]	Mechanisms of Ageing and Development	9	2021
		[4]	International Journal of Environmental Research an	8	2021
		[23]	Maturitas	3	2022
		[112]	Journal of Population Ageing	0	2022
		[9]	American Journal of Lifestyle Medicine	0	2022
	Total N	57	57	57	57

## Appendix C

**Table A3:** Integration of ADE20K annotation format in age-friendly built environment feature for image segmentation.

Category	Annotation Label	Description/Examples	ADE20K

Housing and buildings	Building design: entrance, ground floor, door ways	Entrances without stairs, possibly with ramps or level with the ground, allowing easy access for mobility-impaired individuals. Doorways wide enough to accommodate wheelchairs and walkers. Housing units with ground floor access, minimising the need for stair navigation.	#Door, #door frame, #double door, #stair, #steps, #stairway, #staircase, #window, #window pane, #ceiling
	Housing options		#house, #apartment

Public/open spaces	Third places	Parks, gardens, open spaces that are easily accessible and safe for older adults.	#tree, #flower, #plant, #flora, # plant life, #pot, #flowerpot, #vase, #mountain, #field, #rock, #bush #river, #sea, #water,
	Street furniture		#toilet, #commode, #crapper,
	Pedestrian infrastructure	Pathways with pedestrian-friendly amenities, with adequate lighting, benches, and clear signage.	#light, #light source, #lamp, # street light, #street lamp #chair, #table, #bench #pillar, #edifice, #seat, #sidewalk, #signboard, #sign
	Physical activity	Walking or cycling.	#person, #individual, #someone, #somebody, #bike, #cycling, #route
Transportation systems			
	Public transport access	Available public transport modes like bus, taxi, or train.	#car, #bus, #machine, #autobus, #coach, #truck, van, coach, #charabanc
	Well-designed stations	Bus stops and train stations with features like easy navigation; ramps; elevators; and clear, informational displays.	#busstop, #traffic light, #traffic signal, #stoplight
	Bike paths	Designated bike paths, possibly including electric bicycle charging stations.	#minibike, #bicycle, #bike, #wheel, #cycle, #road, #route, #sidewalk, #pavement, path
